# Exercise rejuvenates the “muscle-heart” crosstalk: skeletal muscle-derived exosomal miRNAs in cardiac aging

**DOI:** 10.3389/fcvm.2026.1863985

**Published:** 2026-07-13

**Authors:** Ruibin Jing, Xue Pang, Xiaona Zhu, Sibao Huang, Xing Gao

**Affiliations:** 1 College of Physical Education, Shandong University of Aeronautics, Binzhou, China; 2 Liaocheng People's Hospital, Liaocheng, China; 3 School of Physical Education Science, Lingnan Normal University, Zhanjiang, Guangdong Province, China; 4 Central People's Hospital of Zhanjiang, Zhanjiang, Guangdong Province, China; 5 Hebei Institute of Communications, Gaoying Street, Chang'an District, Shijiazhuang City, Hebei Province, China

**Keywords:** cardiac aging, exercise mimetics, exerkines, extracellular vesicles (Exosomes), microRNAs (myomiRs), sarcopenia, skeletal muscle-Heart crosstalk

## Abstract

The deleterious intersection of sarcopenia and age-related heart failure represents a profound global health challenge. While skeletal muscle is increasingly recognized as a major endocrine hub, cannot fully account for the persistent epigenetic changes in the aged myocardium. This points to a key mechanistic gap in the “muscle-heart” inter-organ crosstalk. Following PRISMA guidelines, this systematic review (incorporating 51 rigorous *in vivo* and clinical studies) maps the bimodal skeletal muscle-derived extracellular vesicle (SkM-EV) and microRNA (miRNA) axis in cardiac aging. We delineate a pathological baseline where aging and sarcopenia trigger the release of senescence-associated extracellular vesicles (EVs). These toxic vesicular payloads actively propagate myocardial inflammaging, structural remodeling, and apoptosis. Conversely, regular exercise rejuvenates by this network via an epigenetic mechanism. Mechanical loading stimulates the systemic release of “exerkines”—exercise-conditioned EVs enriched with potent cardioprotective myomiRs (e.g., miR-1, miR-133a, miR-342-5p). By systematically categorizing these findings from single-molecule downstream targets (anti-apoptosis, anti-fibrosis) to macroscopic poly-pathway synergy (antioxidant and metabolic reprogramming), we construct a comprehensive molecular roadmap of EV-mediated myocardial rejuvenation. Ultimately, deciphering this vesicular signaling network will elucidate the fundamental epigenetic mechanisms underlying “exercise as medicine,” and paves the way for novel translational horizons. We propose that targeting the bimodal SkM-EV axis will accelerate the development of EV-based liquid biopsies for sarcopenic cardiomyopathy and pioneer cell-free “exercise mimetics” for frail, exercise-intolerant aging populations.

## 1 Introduction

The rapid aging of the global population has made age-related cardiovascular diseases (CVDs) and heart failure leading causes of death and disability worldwide. Clinically, the deterioration of the aging heart rarely occurs in isolation; it overwhelmingly presents alongside age-related loss of skeletal muscle mass and function, a condition termed sarcopenia (1, 2) The deleterious intersection of these two pathologies has given rise to the concept of “sarcopenic cardiomyopathy,” a lethal multi-morbidity characterized by a vicious cycle of systemic frailty and progressive heart failure. Historically, therapeutic strategies have treated the myocardium and the musculoskeletal system as independent entities. However, an emerging biological paradigm suggests that the heart and skeletal muscle are intimately synchronized via profound, bi-directional inter-organ crosstalk, highlighting systemic milieu alteration as a primary driver of cardiac senescence (3, 4).

This paradigm shift recognizes skeletal muscle as not just a locomotive organ, but the body's largest endocrine gland. Under physiological conditions, the muscle continuously secretes a myriad of signaling molecules to maintain systemic homeostasis (5, 6). Crucially, during aging and sarcopenic progression, the skeletal muscle transitions into a systemic “endocrine disruptor.” Senescent myofibers and satellite cells develop a Senescence-Associated Secretory Phenotype (SASP), releasing circulating factors that precipitate distant organ dysfunction (7, 8). Yet, canonical soluble myokines alone are insufficient to fully explain the complex, durable, and targeted epigenetic reprogramming observed in the aging myocardium, underscoring a critical mechanistic gap in our understanding of the muscle-heart axis (9, 10).

Recent breakthroughs in intercellular communication have bridged this gap by identifying extracellular vesicles (EVs), particularly exosomes, as key mediators of inter-organ signaling (11). These lipid bilayer nanovesicles encapsulate a highly specific cargo of proteins, lipids, and—most importantly—microRNAs (miRNAs). Protected from enzymatic degradation, skeletal muscle-derived EVs (SkM-EVs) traverse the systemic circulation and are directly internalized by cardiomyocytes, fibroblasts, and endothelial cells (10, 12). Existing evidence implies a bimodal nature of SkM-EVs: in the aged state, they act as pathological vectors, delivering senescence-inducing and pro-fibrotic miRNAs that actively propagate cardiac inflammaging (7, 13). For instance, muscle-derived miR-34a has been shown to increase with age in circulating EVs and to induce senescence in recipient stem cells, while disuse atrophy alters the miRNA cargo of fibro-adipogenic progenitor EVs to favor senescence and atrophy pathways (13–15). Conversely, regular mechanical loading (physical exercise) acts as a robust epigenetic switch, triggering the release of “exerkines”—exercise-conditioned EVs enriched with potent antioxidant and anti-apoptotic payloads capable of reversing structural myocardial remodeling (16–18). This protective vesicular signature is further underscored by findings that EVs from exercised muscle can attenuate fibrotic and inflammatory injury in distant organs such as the kidney,underscoring that muscle-derived signals have systemic reach (19).

Despite the explosive growth of independent preclinical investigations into exercise, exosomes, and cardiac aging, a comprehensive and structured synthesis of this complex bimodal network is notably absent from current literature (20, 21). Recently, an array of excellent reviews has elegantly summarized the endocrine role of skeletal muscle. These works have primarily mapped SkM-EVs' crosstalk with adipose tissue regarding systemic metabolism (22), explored the muscle-bone axis in the context of osteosarcopenia (23), highlighted their emerging roles in glucose and lipid metabolic regulatio (24), or provided a broad overview of EV-mediated communication across multiple systemic organs (25). Parallelly, the generalized concept of integrative cardiac exercise rehabilitation has also been comprehensively reviewed (26). However, the specific bidirectional ‘muscle-heart vesicular axis’ in cardiac aging has not been systematically examined. This review addresses this gap by contrasting. Therefore, differentiating from and expanding upon the existing macroscopic literature, this systematic review specifically interrogates the bimodal paradigm—contrasting the pathological baseline of sarcopenia-induced cardiac inflammaging against the restorative mechanisms of exercise-rejuvenated EVs. By exclusively focusing on myocardial senescence, we aim to construct a precision molecular roadmap that paves the way for heart-specific EV-based liquid biopsies and cell-free “exercise mimetics”.

Therefore, this systematic review was conducted in strict accordance with the PRISMA guidelines to dynamically map the “muscle-heart” bimodal vesicular axis. By interrogating the pathological baseline of the sarcopenia-heart crosstalk —exemplified by the transfer of atrophic muscle-derived miR-125a-5p and miR-690 to suppress osteogenesis and satellite cell differentiation, respectively (16, 27)—and contrasting it with the restorative mechanisms of SkM-EVs induced by exercise, this review aims to construct a molecular roadmap of exosomal miRNAs targeting myocardial senescence. Furthermore, understanding how muscle-derived EVs mediate systemic aging, such as the transfer of miR-29b-3p to neuronal cells or the aggravation of kidney injury via miR-21a-3p, provides a broader context for the muscle-heart axis (19, 28). Ultimately, unlocking this vesicular language will not only elucidate the epigenetic mechanisms behind “exercise as medicine,” but also pave the way for novel translational horizons, including EV-based liquid biopsies and cell-free “exercise mimetics” for the aging population (18, 29).

## 2 Methodology: systematic literature search strategy and selection criteria

To ensure a rigorous synthesis of the current evidence regarding the skeletal muscle-heart axis, this review was conducted following a structured methodological framework. The literature search, study selection, and data extraction processes were rigorously designed to capture state-of-the-art discoveries concerning exercise-induced extracellular vesicles (EVs) and their epigenetic cargos.

### 2.1 Search strategy and databases

A comprehensive electronic literature search was executed across three primary databases: PubMed, Web of Science (Core Collection), and Scopus, covering literature published from January 2010 through March 2026. To fully capture the complexity of sarcopenic cardiomyopathy and inter-organ communication, we employed a tripartite Boolean search algorithm utilizing a combination of Medical Subject Headings (MeSH) and free-text keywords:

Domain 1 (Pathological Baseline: Sarcopenia-Heart Axis): Focused on the pathological link between muscle wasting and cardiac dysfunction. Search terms combined sarcopenia-related keywords: (“sarcopenia” OR “muscle atrophy” OR “aging muscle” OR “skeletal muscle aging”) AND (“heart failure” OR “cardiomyopathy” OR “myocardial remodeling” OR “cardiac aging”).

Domain 2 (Mechanistic Link: Senescence-Associated Exosomes): Targeted the mechanistic role of senescence-associated exosomes. We used terms for extracellular vesicles: (“exosome” OR “extracellular vesicle*”) AND (“aging” OR “senescence” OR “SASP” OR “inflammaging”) AND (“microRNA” OR “miRNA”) AND “skeletal muscle”.*

Domain 3 (Therapeutic Intervention: Exercise Rejuvenation): Captured the therapeutic effects of exercise. Search terms included exercise modalities: (“exercise” OR “resistance training” OR “aerobic training”) AND (“exosome*” OR “extracellular vesicle*”) AND (“aging” OR “senescence” OR “sarcopenia”) AND (“skeletal muscle” OR “myomiR*”)

All retrieved citations were imported into a reference management software (e.g., EndNote), and duplicates were automatically removed.

### 2.2 Inclusion and exclusion criteria

We used the PICOS framework to define inclusion criteria (30); (1) Population/Models: studies utilizing natural aging *in vivo* models (e.g., aged rodents) or older human cohorts (>60 years); (2) Intervention: involving any standardized *in vivo* exercise protocol (e.g., aerobic or resistance training); (3) Targets: explicitly investigating skeletal muscle-derived exosomes/sEVs and their miRNA payloads; (4) Outcomes: documented effects on senescence markers, autophagy, mitochondrial homeostasis, or myocardial remodeling; and (5) Study Design: peer-reviewed original research published in English.

**Figure 1:**
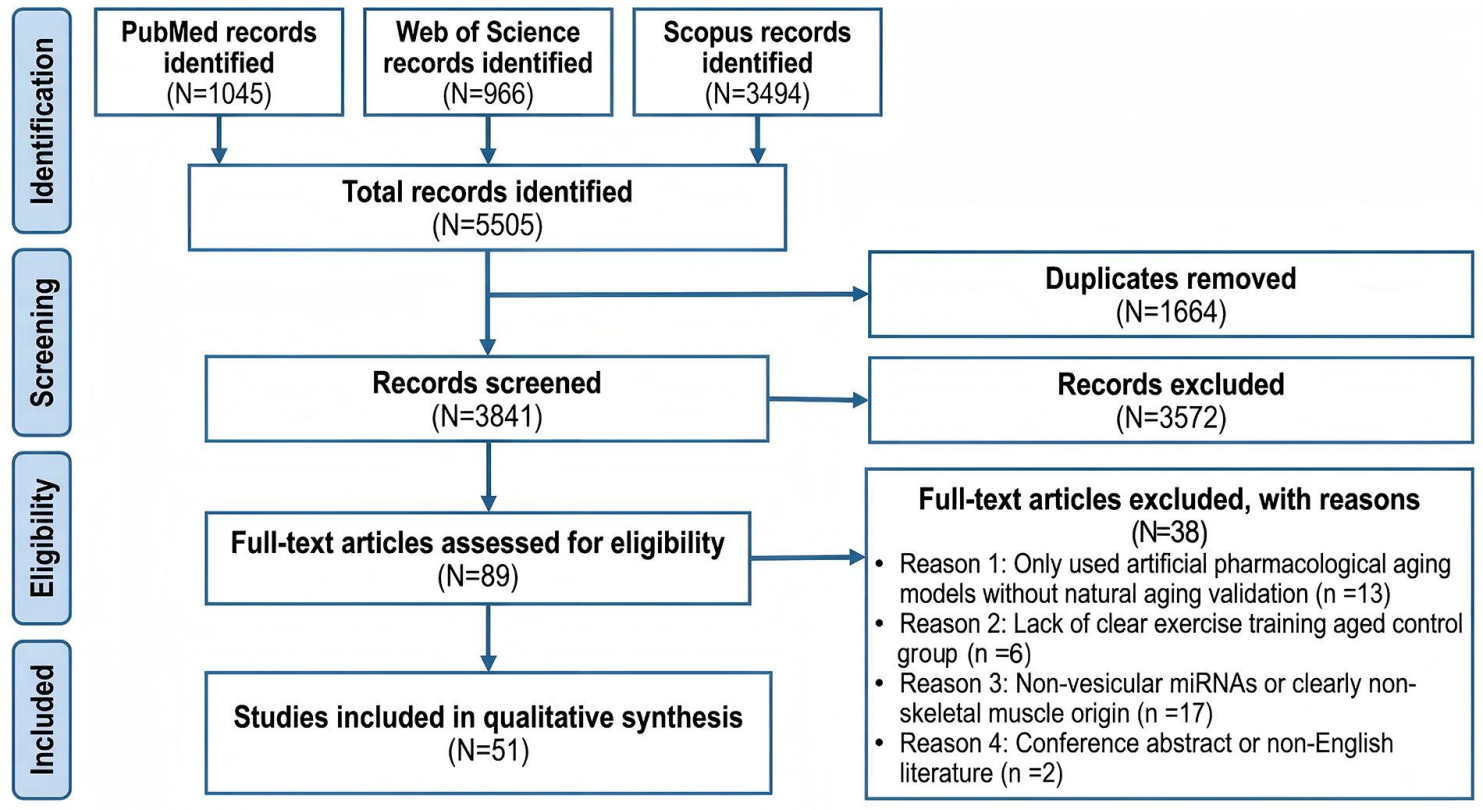
PRISMA 2020 flow diagram detailing the systematic literature search and study selection process. A comprehensive electronic search across PubMed, Web of Science, and Scopus yielded an initial total of 5,505 records. Following the automatic removal of 1,664 duplicates, 3,841 titles and abstracts were independently screened. Subsequently, 89 full-text articles were critically assessed for eligibility based on predefined PICOS criteria. Of these, 38articles were excluded due to specific methodological deviations as listed. Ultimately, 51 original peer-reviewed studies met all criteria and were included in the qualitative synthesis of this bimodal “muscle-heart” axis review.

Conversely, literature was excluded based on the following criteria: (1) studies solely utilizing artificial pharmacological aging models (e.g., D-galactose induction) without validation in naturally aged models; (2) interventions lacking an explicit exercise-trained aged control group; (3) research investigating non-vesicular circulating miRNAs or exosomes unequivocally derived from non-muscle origins; and (4) case reports, conference abstracts, or non-English publications. Two independent reviewers screened the titles and abstracts, followed by a full-text critical appraisal of the eligible articles to extract multidimensional mechanistic data. The detailed study selection process, including the precise number of records identified, screened, critically appraised, and ultimately included, is visually summarized in the PRISMA flow diagram (Figure 1),which was generated in compliance with the PRISMA 2020 statement (31) using a validated interactive tool (32).

### 2.3 Data extraction and synthesis

Given the highly mechanistic and molecular focus of this review, a qualitative and thematic synthesis approach was adopted rather than a quantitative meta-analysis. To rigorously construct the biological narrative based on the included 51 studies, the data extraction process was executed in three sequential stages, resulting in three distinct, categorized data matrices (spreadsheets) that correspond to the core chapters of this manuscript.

Specifically, data were systematically extracted and synthesized as follows:

- Stage 1: Establishing the Bimodal Baseline and Pathological Crosstalk (Corresponding to Chapter 3).
  In the first data matrix, extraction focused on the divergent physiological states of skeletal muscle (e.g., aging, sarcopenia, cachexia vs. basal health). Key variables tabulated included the exact origin of pathogenic vesicles [e.g., mitochondria-derived vesicles (MDVs) or SASP-related EVs], the altered “toxic” payloads (e.g., mtDNA, miR-34a, let-7), and the profound depletion of constitutive protective factors (e.g., MyomiRs such as miR-1 and miR-133a). This matrix synthesized the underlying pathological baseline of the bimodal muscle-heart axis.
- Stage 2: Mapping Specific Molecular Targets of Exercise-EVs (Corresponding to Chapter 4).
  The second spreadsheet focused on specific mechanistic pathways linking skeletal muscle to the heart. For studies using exercise or mechanical loading models, we extracted the exercise paradigm, the reported cardioprotective cargoes, the target cell types in the heart, the downstream signaling pathways, and the resulting phenotypic outcomes. Examples included swimming and treadmill running; cargoes such as miR-342-5p, miR-130a, the miR-29b cluster, and HSP70; target cells including cardiomyocytes and endothelial cells; signaling pathways such as Caspase 9/JNK2, MMP9 inhibition, and ROS/NF-*κ*B; and outcomes including anti-apoptotic, anti-fibrotic, and angiogenic effects.
- Stage 3: Delineating Macroscopic Networks and Synergy (Corresponding to Chapter 5).
  To elevate the analysis beyond reductionist “single-molecule” views, the third matrix aggregated data reflecting systemic, multi-cellular interactions. Extraction here focused on overlapping signaling hubs (e.g., PI3 K/Akt, MAPK, TGF-*β*), systemic antioxidant capacities (e.g., SOD2, Nrf2), and broader inter-organ metabolic reprogramming. This facilitated the conceptualization of the “polypharmacy” effect, illustrating how exercise-derived myomiR cocktails synergistically ameliorate myocardial remodeling.
  Ultimately, these three constructed data matrices served as the foundational scaffolding for the narrative synthesis. Rather than statistical pooling, this thematic categorization allowed for the precise deciphering of complex intracellular signaling cascades, seamlessly bridging the gap between basic bimodal biology and advanced cell-free therapeutic concepts. Each matrix was not only used for data storage, but also served as the analytic scaffold for the corresponding chapter. Specifically, recurring variables, convergent pathways, and shared phenotypic outputs within each matrix were iteratively grouped into higher-order mechanistic themes, which then became the subsection structure of Chapters 3, 4, and 5. To explicitly delineate the path from raw data extraction to narrative synthesis, an analytical crosswalk linking the extraction variables to the manuscript's structural output is presented in Table 1.

**Table 1:** Analytical crosswalk linking the three extraction matrices to the narrative synthesis structure of chapters 3–5.

Stage	Corresponding Chapter	Extraction Focus	Output
Stage 1	Chapter 3	Vesicle origin, pathogenic/protective cargo, pathological baseline	Pathological crosstalk and loss of shield
Stage 2	Chapter 4	Exercise paradigm, EV cargo, target cell, pathway, phenotype	Specific cardioprotective mechanisms
Stage 3	Chapter 5	Shared signaling hubs, systemic interactions, translational synergy	Biomarkers and exercise mimetics

## 3 The bimodal skeletal muscle-heart axis: From pathological crosstalk to exercise-induced cardioprotection

Drawing upon the first data matrix generated in Stage 1 of our methodology (Section 2.3), this section systematically contrasts the divergent physiological states of skeletal muscle. The synthesis below translates the tabulated variables—specifically the origins of pathogenic vesicles, their altered payloads, and the depletion of protective factors—into a mechanistic narrative of the bimodal muscle-heart axis.

### 3.1 Introduction: skeletal muscle as an endocrine organ and the mechanical trigger of vesicular secretion

Beyond its roles in locomotion and metabolism, skeletal muscle (SkM) is now recognized as a major endocrine organ. It orchestrates systemic homeostasis by actively secreting a plethora of bioactive molecules into the circulation, collectively termed “myokines.” (33) However, recent advances in transcriptomic and proteomic profiling have catalyzed a paradigm shift, revealing that classical soluble myokines represent only a fraction of the muscle's secretome. The critical vectors for high-fidelity, long-distance inter-organ communication—particularly within the “muscle-heart” axis—are skeletal muscle-derived extracellular vesicles (SkM-EVs), predominantly exosomes (34, 35). These lipid bilayer-enclosed nanovesicles confer exceptional structural stability to their cargo, enabling the targeted delivery of regulatory microRNAs (miRNAs) and functional proteins to distant myocardial tissues, thereby escaping systemic enzymatic degradation (36). The foundational basis for this vesicular crosstalk was established by pioneering studies identifying that skeletal muscle cells actively secrete exosomes enriched with specific miRNAs, which function as potent endocrine signals to regulate gene expression in recipient cells (37). Furthermore, the profound dysregulation of these evolutionary conserved miRNAs has long been recognized as a core pathophysiological driver of sarcopenia and age-related muscle homeostasis disruption (38).

Crucially, the biogenesis and systemic release of SkM-EVs are not merely constitutive basal processes; rather, they are exquisitely responsive to physiological stimuli, primarily mechanical stress (39). At the molecular level, physical exertion initiates a mechanotransduction cascade via the activation of focal adhesion integrins, mechanosensitive calcium channels, and primary cilia located on the sarcolemma (40). This mechanosensory signaling provokes a robust intracellular calcium influx, which acts as the fundamental catalyst for the fusion of multivesicular bodies (MVBs) with the plasma membrane and the subsequent exocytosis of EVs (41). Consequently, exercise induces a dramatic, transient surge in circulating EVs—often eliciting a greater than two-fold increase in microvesicles within hours post-exercise (42, 43).

Concurrently, this mechanical stress triggers an acute upregulation in the transcription and vesicular packaging of pleiotropic factors. The exercise-mobilized SkM-EVs are significantly enriched with mRNAs and secreted proteins, including interleukin-6 (IL-6), insulin-like growth factor 1 (IGF-1), CYR61/CCN1, and ANGPTL4 (44). This calcium-dependent EV mobilization provides the direct mechanistic basis for how macroscopic physical exertion is translated into microscopic, vesicle-mediated inter-organ crosstalk (45).

However, this SkM-EV-mediated network operates as a highly sensitive bimodal axis, strictly contingent upon the physiological or pathological state of the source muscle (46). While mechanical loading (exercise) actively enriches SkM-EVs with anti-apoptotic and pro-angiogenic cargos (e.g., IGF-1 and cardioprotective miRNAs), the absence of mechanical stimuli (disuse) or the onset of muscular senescence fundamentally distorts this vesicular payload (47). The transition from a mechanically stimulated SkM secretome to an aged, maladaptive one is central to this bimodal axis. It shifts the network from cardiovascular maintenance toward pathological crosstalk, thereby driving cardiac aging and vulnerability (48).

### 3.2 The pathological crosstalk: maladaptive signaling from senescent or atrophic muscle

The skeletal muscle-heart axis is highly vulnerable to the loss of physiological homeostasis. With advancing age, physical inactivity (disuse), or cachexia, skeletal muscle undergoes profound phenotypic alterations, shifting from a cardioprotective endocrine organ to a primary source of systemic toxicity (49). This maladaptive transition is largely mediated by a dramatic reconfiguration of the SkM-EV cargo, which actively propagates senescence and metabolic stress to the highly perfused myocardium (50).

#### 3.2.1 Pathological myokines and the senescence-associated secretory phenotype (SASP)

In the context of sarcopenia and biological aging, triggers the senescence-associated secretory phenotype (SASP), characterized by the robust secretion of pro-inflammatory cytokines (24). As delineated by Kamal et al., this aged SkM secretome—heavily loaded with pro-inflammatory cytokines (IL-6, TNF-α) and pro-fibrotic factors (TGF-*β*)—enters the systemic circulation via EVs, driving chronic “inflammaging” and exerting direct detrimental effects on cardiac resident cells (23).

Beyond classical SASP proteins, the miRNA transcriptome of aged SkM-EVs is fundamentally corrupted (51). Mapping back to our Stage 1 extraction variables (Section 2.3), this pathological crosstalk can be decoded into three distinct mechanistic components:

(1) Altered Pathogenic Cargo: Transcriptomic profiling of EVs derived from senescent or stressed skeletal muscle reveals a distinct “pathogenic payload” characterized by the profound upregulation of specific miRNAs (52). For instance, studies by Fulzele et al. and Qin et al. have identified miR-34a as a canonical aging marker significantly enriched in muscle-derived EVs under oxidative stress (53).
(2) Specific Signaling Targets: Upon entering circulation, these cargos actively target critical homeostatic regulators. Circulating miR-34a, for instance, actively downregulates SIRT1 (Sirtuin 1), a master anti-aging deacetylase (14).
(3) Distal Cardiac Consequences: Similarly, models of disuse atrophy (immobilization) and cachexia have demonstrated the massive release of senescence-associated miRNAs, including the let-7 family, miR-181a and miR-21a-3p (54). While these circulating pathogenic EVs extensively disrupt homeostasis in various distal organs (e.g., exacerbating renal fibrosis and disrupting osteogenesis), the myocardium, due to its immense vascular density and metabolic demand, is highly susceptible to the prominent uptake and accumulation of these circulating fibrotic and apoptotic cues, accelerating accelerating age-related myocardial remodeling and extracellular matrix stiffening (55).

#### 3.2.2 Systemic metabolic dysregulation and mitochondria-derived stress signals

A hallmark of skeletal muscle aging is the progressive decline in mitochondrial quality control (55). Given that skeletal muscle accounts for roughly 40% of body mass and houses a massive mitochondrial network, its mitochondrial dysfunction has catastrophic systemic implications (56). Recent groundbreaking evidence, particularly highlighted by Marzetti et al., has expanded the paradigm of vesicular crosstalk by identifying Mitochondria-Derived Vesicles (MDVs) as potent mediators of systemic metabolic dysregulation (4).

In aged and frail skeletal muscle, impaired mitophagy leads to the accumulation of damaged mitochondrial components. To alleviate local stress, muscle cells package these damaged constituents—specifically mitochondrial DNA (mtDNA) and oxidized mitochondrial proteins—into MDVs and small EVs, expelling them into the extracellular space (57). Upon entering the systemic circulation, these mtDNA-containing EVs function as potent Damage-Associated Molecular Patterns (DAMPs). They are recognized by pattern recognition receptors in distal tissues, triggering profound sterile inflammation primarily via the activation of the cGAS-STING (cyclic GMP-AMP synthase-stimulator of interferon genes) pathway (58). For the aging heart, which already suffers from diminished metabolic flexibility, the continuous bombardment by SkM-derived MDVs and circulating DAMPs severely disrupts myocardial energy homeostasis, amplifies intracellular oxidative stress, and significantly contributes to age-related cardiac dysfunction (59).

Collectively, the age- or atrophy-induced inversion of the SkM-EV profile establishes a toxic systemic milieu. This pathological crosstalk serves as a previously underappreciated systemic driver of cardiovascular aging, providing a mechanistic rationale for the intimate clinical correlation between sarcopenia and heart failure (60).

### 3.3 The loss of homeostatic defense: depletion of protective skeletal muscle-derived EVs (SkM-EVs) in disease states

The pathological progression of cardiovascular aging is not solely driven by the active secretion of detrimental SASP factors (as discussed in Section 2.2); it is equally precipitated by a profound “loss of function” within the bimodal SkM-heart axis (61). Under homeostatic physiological conditions, healthy skeletal muscle maintains a basal, constitutive secretion of protective SkM-EVs. This vesicular pool serves as an essential endocrine buffering system, continuously supplying the myocardium with anti-apoptotic, pro-angiogenic, and metabolism-regulating cargo. However, during the progression of sarcopenia or chronic heart failure (CHF), this protective vesicular repertoire undergoes significant depletion, rendering the aging heart defenseless against cumulative stress (62).

### 3.4 The baseline Myo-miRNA repertoire

Mechanistic studies have elegantly defined the physiological baseline of SkM-EVs. As highlighted by Murphy et al., healthy and regenerating skeletal muscle actively secretes EVs highly enriched with muscle-specific microRNAs, termed “MyomiRs” (e.g., miR-1, miR-133a/b, and miR-206) (63). Under normal physiological crosstalk, these circulating MyomiRs are robustly internalized by distal cardiac resident cells, where they exert strict epigenetic control over extracellular matrix (ECM) remodeling, mitigating spontaneous fibrotic responses (64). Furthermore, as demonstrated by Yedigaryan et al., the physiological SkM secretome contains pro-regenerative EV cargos (such as miR-1 and miR-208a) that inherently promote muscle hypertrophy and concurrently act to suppress fibrosis *in vivo*, establishing an anti-fibrotic systemic baseline (62).

### 3.5 Depletion and decompensation

Biological aging of skeletal muscle alters this protective cargo signature. Comprehensive reviews, such as the framework provided by Chung et al., emphasize that physiological aging severely reduces the beneficial components within SkM-EVs (63). The age-induced decline fundamentally depletes the circulating levels of essential cardioprotective cargo, including protective enzymatic mediators (e.g., eNAMPT) and critical MyomiRs (e.g., miR-126 and miR-133a), alongside a significant reduction in the packaging of cytoprotective heat shock proteins (e.g., HSP70) (65, 66).

The pathophysiological consequences of this vesicular depletion are profound for the aging heart. For instance, the sharp decline in circulating SkM-derived miR-126 deprives the cardiac microvasculature of a critical pro-angiogenic signal, thereby accelerating endothelial senescence and capillary rarefaction—key hallmarks of age-related heart failure with preserved ejection fraction (HFpEF) (67). Concurrently, the loss of circulating SkM-derived miR-133a lifts the constitutive inhibition on cardiac fibroblasts, facilitating unbridled collagen deposition and myocardial stiffness (68).

Therefore, the systemic depletion of these protective SkM-EVs represents a critical decompensatory tipping point in cardiovascular aging. It creates a state of systemic vulnerability, defining the exact pathological “baseline” upon which the therapeutic necessity of exercise—or “exercise-rejuvenated” SkM-EVs—is predicated. The identification of this depleted physiological repertoire (e.g., the loss of specific MyomiRs) provides precisely the foundational rationale for future therapeutic interventions (69).

### 3.6 The restorative mechanism: exercise-induced EV reprogramming and precision therapeutic niches

In striking contrast to the maladaptive shedding of pathogenic EVs during aging or inactivity, physical exercise acts as a potent physiological stimulus that fundamentally rejuvenates the “muscle-heart” crosstalk (70). Rather than merely increasing the absolute quantity of circulating vesicles, exercise induces a profound transcriptomic and proteomic reprogramming of the SkM-EV payload (71). This exercise-conditioned secretome is actively enriched with a tailored consortium of cardioprotective miRNAs and antioxidant proteins, which synergistically target distinct myocardial compartments to reverse age-related deterioration and establish precise therapeutic niches (72, 73).

#### 3.6.1 Niche 1: direct myocardial protection (anti-apoptotic and anti-fibrotic cascades)

Exercise-rejuvenated SkM-EVs directly protect cardiomyocytes against lethal ischemic and age-related insults. Pioneering work by Hou et al. demonstrated that long-term exercise (e.g., swimming or treadmill running) significantly upregulates exogenous miR-342-5p in circulating SkM-EVs (74). Upon targeted internalisation by ischemic cardiomyocytes, EV-derived miR-342-5p acts as a potent survival signal, directly targeting and inhibiting the pro-apoptotic mediators Caspase 9 and Jnk2, thereby abrogating mitochondria-dependent intrinsic apoptosis (75). Similarly, Bei et al. showed that exercise training prompts the robust vesicular packaging of cardioprotective proteins, notably Heat Shock Protein 70 (HSP70) (75). The delivery of these HSP70-enriched EVs activates canonical pro-survival kinase cascades (ERK1/2, p38 MAPK, and Akt) in the recipient myocardium, reducing infarct size following ischemia/reperfusion injury (76).

Furthermore, exercise-derived EVs possess striking anti-fibrotic capabilities (3). Chaturvedi et al. identified a distinct exercise-induced vesicular miRNA cluster (comprising miR-455, miR-29b, miR-323-5p, and miR-466) present in both serum and heart tissue following swimming protocols (6). In diabetic and stressed murine models, the synergistic delivery of this miRNA cluster robustly targets and suppresses matrix metalloproteinase-9 (MMP9) expression, effectively halting adverse extracellular matrix (ECM) remodeling and reversing established myocardial fibrosis (77).

#### 3.6.2 Niche 2: endothelial rejuvenation and microvascular resilience

Simultaneously, the therapeutic reach of exercise-conditioned SkM-EVs extends deeply into the cardiac microvasculature (78). As elucidated by Nie et al., myotube-derived EVs post-exercise are highly enriched with miR-130a (71). The unidirectional transfer of miR-130a into cardiac endothelial cells (ECs) actively modulates ROS-mediated NF-*κ*B signaling pathways, potently stimulating EC proliferation, migration, and subsequent angiogenesis (79). This microvascular rejuvenation is further supported by the neutralization of local oxidative stress. Kargl et al., utilizing an exercise-mimetic model (PGC-1*α* overexpression), revealed that SkM-EVs are uniquely loaded with primary antioxidant mRNAs (such as SOD2, Nrf2, and GPx) (80). The horizontal transfer of these transcripts into neighboring endothelial cells exponentially enhances their intracellular ROS-buffering capacity, resisting oxidative stress-induced endothelial senescence (81). Finally, scaling up to *in vivo* atherosclerosis models (ApoE-/- mice), Wang et al. confirmed that exercise-trained EVs suppress the endothelial expression of pro-inflammatory adhesion molecules, stabilizing vascular plaques and alleviating systemic atherosclerotic burdens (82).

**Figure 2:**
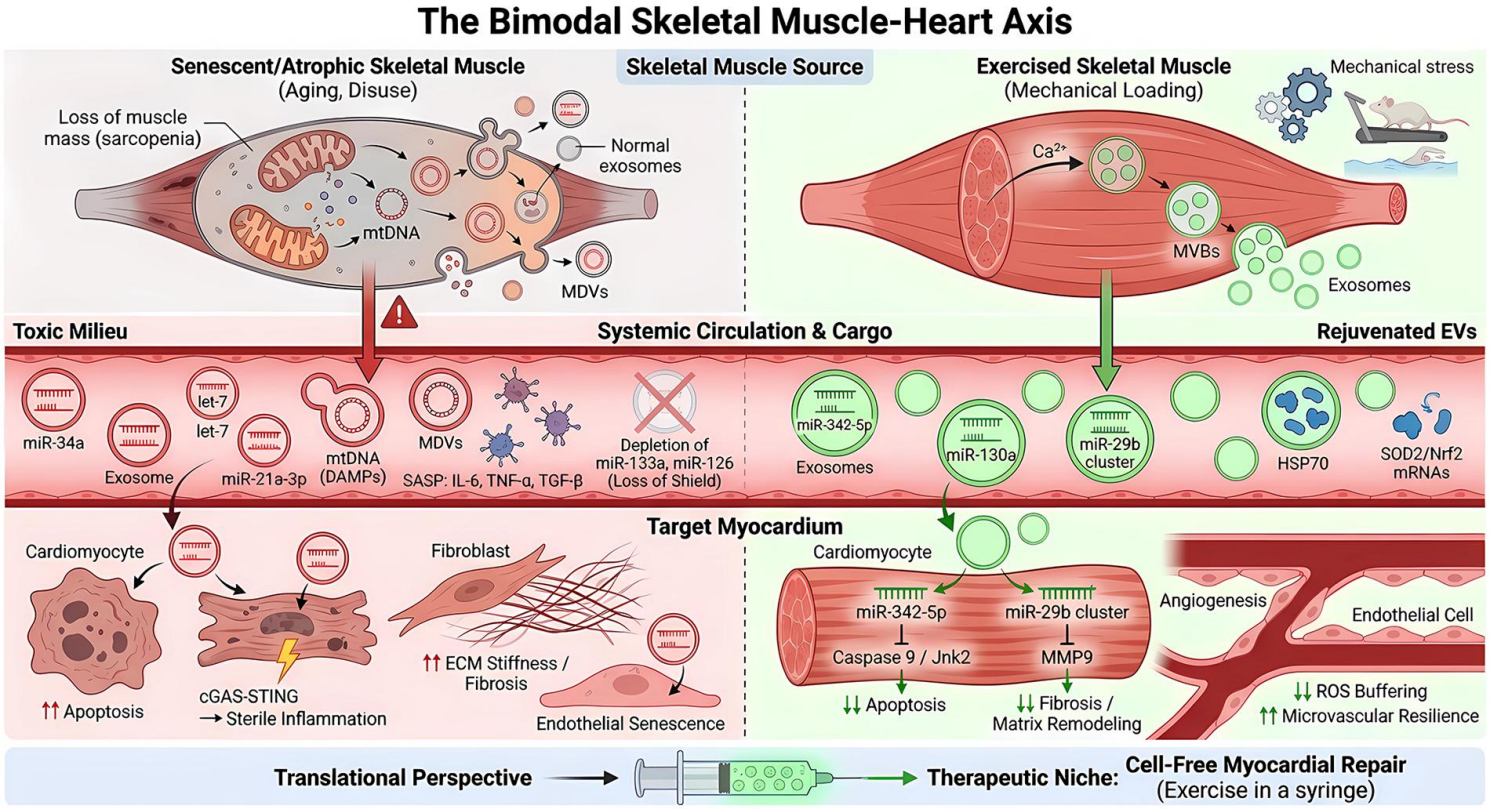
The bimodal SkM-heart axis in aging and exercise. In sarcopenia/aging (Left), senescent muscle releases pathogenic EVs (e.g., MDVs with mtDNA, exosomes with miR-34a) while depleting protective myomiRs, promoting cardiac inflammation, apoptosis, and fibrosis. In response to exercise (Right), muscle releases rejuvenated EVs enriched with cardioprotective miRNAs (e.g., miR-342-5p, miR-130a) and antioxidant mRNAs, which inhibit apoptosis, reverse fibrosis, and promote angiogenesis in the aging heart. (*Created with BioRender.com*). SkM, skeletal muscle; EVs, extracellular vesicles; MDVs, mitochondria-derived vesicles; mtDNA, mitochondrial DNA; SASP, senescence-associated secretory phenotype; DAMPs, damage-associated molecular patterns; ECM, extracellular matrix; ROS, reactive oxygen species*.*

Collectively, this bimodal paradigm—visually synthesized in Figure 2—reveals that exercise not only provides systemic benefits but also precisely reprograms SkM-EVs for targeted myocardial repair. By elucidating how exercise reprograms SkM-EVs to explicitly target cardiomyocyte survival, microvascular expansion, and oxidative buffering, we obtain the exact molecular blueprints required for myocardial repair. The specific molecular cargos identified here define the specific “therapeutic niches” that modern cell-free engineering therapies seek to exploit, paving the way for advanced interventional strategies (28, 83).

### 3.7 Summary and translational perspectives: paving the Way for cell-free myocardial repair

As conceptualized in Figure 2, the current body of evidence elucidates a profound bimodal dichotomy within the skeletal muscle-heart bimodal axis. Specifically, the top panel of Figure 2 visually maps the maladaptive shift: biological aging and physical disuse actively drive a pathological EV secretome, bombarding the highly perfused myocardium with toxic SASP components and senescence-associated miRNAs, while simultaneously stripping the heart of its constitutive vesicular defense (84). In stark contrast, the lower panel of Figure 2 illustrates the therapeutic countermeasure: physical exercise acts as a robust physiological switch, orchestrating a comprehensive reprogramming of EV biogenesis to deploy precise, cardioprotective molecular payloads (e.g., miR-342-5p, miR-130a, and the miR-29b cluster) that directly rescue ischemic cardiomyocytes and rejuvenate microvascular networks (85).

However, recognizing this bimodal mechanism reveals a fundamental clinical paradox: the patient populations most acutely vulnerable to cardiovascular aging and heart failure—specifically the frail, the elderly, and those with advanced cachexia or sarcopenia—often suffer from profound exercise intolerance (86). For these critically ill demographics, achieving the intensity of mechanical loading required to naturally mobilize protective SkM-EVs is clinically unfeasible (87).

This inherent limitation of physical rehabilitation necessitates a paradigm shift toward translational bioengineering and “cell-free” therapeutic modalities. By rigorously decoding the molecular blueprints of exercise-conditioned SkM-EVs, we transition from merely observing physiological phenomena to actively designing interventional strategies (88). The specific therapeutic niches identified in Section 2.4 provide the exact biomolecular templates required for innovation. Consequently, a compelling translational frontier emerges: Can we systematically harvest natural exercise-conditioned EVs, or leverage cutting-edge nanotechnology to engineer naive exosomes loaded with these definitive cardioprotective MyomiRs? By uncoupling the molecular benefits of exercise from the mechanical act of exertion itself, we hold the potential to deliver the cardiovascular equivalent of “exercise mimetics.” (89) The feasibility, methodologies, and translational challenges of these advanced, EV-based myocardial repair strategies will be systematically explored in Chapter 4 (90).

## 4 Exercise-Rejuvenated skeletal muscle EVs: therapeutic targets in the aging heart

**Figure 3:**
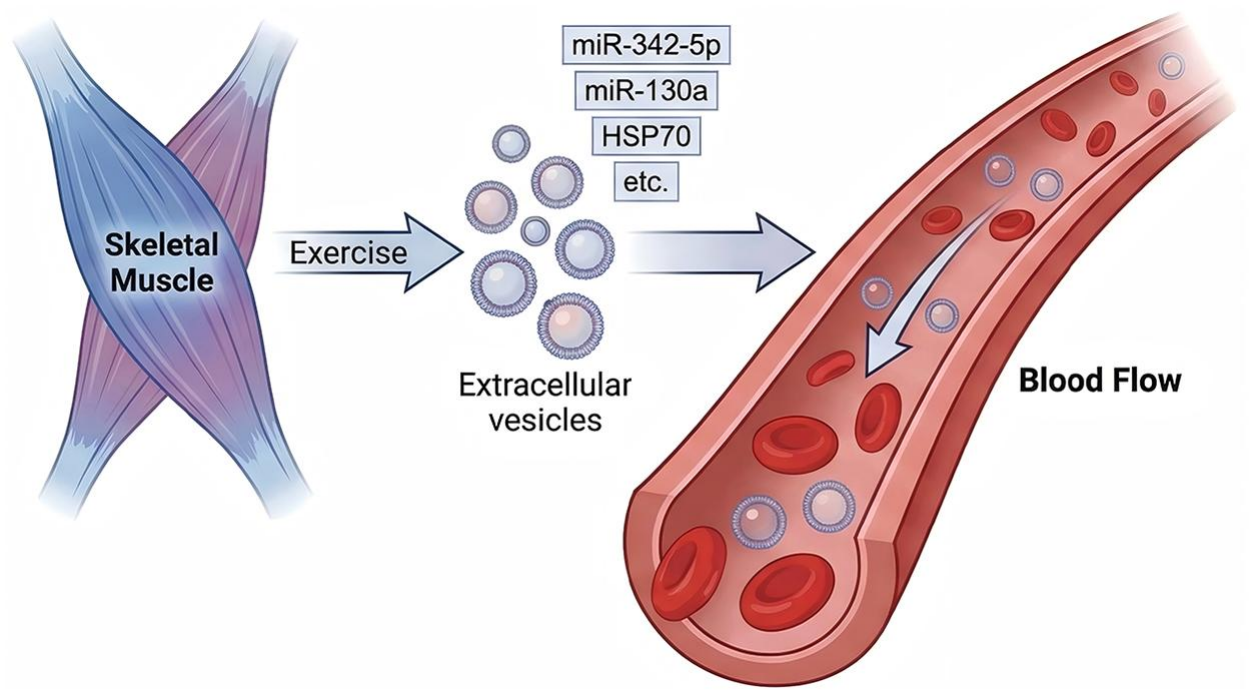
Mechanisms of exercise-rejuvenated “muscle-heart” crosstalk mediated by skeletal muscle-derived extracellular vesicles (SkM-EVs). Exercise stimulates the release of SkM-EVs carrying cardioprotective miRNAs (miR-342-5p, miR-130a) and HSP70. These EVs target both cardiomyocytes (inhibiting Caspase 9/Jnk2-mediated apoptosis and MMP9-driven fibrosis) and endothelial cells (reducing ROS and promoting NF-*κ*B-mediated angiogenesis), thereby reversing age-related cardiac decline. (Created with BioRender.com). SkM-EVs, skeletal muscle-derived extracellular vesicles; ECM, extracellular matrix; MMP9, matrix metalloproteinase 9; ECs, endothelial cells; ROS, reactive oxygen species.

Building upon the bimodal paradigm established above, the precise orchestration of this “muscle-heart” rejuvenation relies on specific molecular effectors. As summarized in Figure 3, Physical exertion actively mobilizes SkM-EVs, acting as an epigenetic trigger for the selective packaging of critical microRNAs (e.g., miR-342-5p, miR-130a) and stress-response proteins (e.g., HSP70) (91). Through the circulatory system, these mobilized EVs accurately hone in on the cardiac microenvironment. To explicitly illustrate this downstream targeting, Figure 3 visually separates this pleiotropic rejuvenation into two distinct anatomical compartments: one pathway details how EVs directly preserve cardiomyocytes against apoptosis (via Caspase 9 inhibition) and fibrosis, while the parallel pathway outlines how EVs concomitantly revive the endothelial cells (ECs) via enhanced NF-*κ*B-mediated angiogenesis and redox homeostasis (92).

### 4.1 Direct protection of cardiomyocytes: anti-apoptosis and anti-fibrosis

Cardiomyocyte loss via apoptosis and the progressive stiffening of the myocardium due to excessive extracellular matrix (ECM) deposition (fibrosis) are two primary hallmarks of cardiac aging and metabolic stress (93). Exercise-induced circulating EVs directly protect the heart. As crucial mediators of muscle-heart crosstalk, they deliver specific molecular cargos that antagonize cardiomyocyte apoptosis and fibrosis. Directly derived from the data extraction in Stage 2 (mapping specific molecular targets), Table 2 presents the formalized data matrix. This matrix systematically isolates the unidirectional mechanistic axes, linking exact exercise models to specific EV cargos, target cells, signaling pathways, and phenotypic outcomes (94, 95).

**Table 2:** Data matrix synthesis (stage 2): characteristics and cardioprotective mechanisms of exercise-derived skeletal muscle extracellular vesicles (SkM-EVs) in aging and pathological models.

Model	EV Origin	Key Cargo Altered	Target Cell In Heart	Signaling Pathway	Cardiac Phenotype	Ref
Mice, 3-Wk swimming	Post-Exercise plasma	↑ EV number; ↑ HSP70 & other proteins	Cardiomyocytes	Activation of ERK1/2, p38 MAPK, and Akt	Attenuated I/R injury; reduced infarct size; inhibited apoptosis	(98)
db/db mice+8-wk swimming	Post-Exercise heart tissue & serum	Upregulation of miR-455, miR-29b, miR-323–5p, miR-466	Cardiomyocytes	Targeted inhibition of MMP9	Attenuated diabetic fibrosi; improved ECM remodeling	(102)
Rowers (1-yr training)/Mice,4-wk swimming	Post- Exercise plasma	Significantupregulation of miR-342-5p	Cardiomyocytes	Inhibition of Caspase9 and Jnk2	Suppressed mitochondrial apoptosis; Reduced ischemic injury.	(74)
Primary human myotubes (PGC-1*α* overexpression)	Myotube- derived EVs (SkM-EVs)	Enrichment of antioxidant mRNAs (SOD2, Nrf2, GPx)	Endothelial Cells (HUVECs)	Enhanced Intracellular ROS buffering	Promoted angiogenesis; resisted oxidative stress- inducedsenescence.	(70)
C2C12 myotubes	Conditioned medium from differentiated myotubes	High enrichment of miR-130a	Endothelial cells(HUVECs)	ROS-mediated NF-*κ*B signaling	Promoted EC Proliferation, migration, and tube formation (angiogenesis).	(71)
ApoE-/- mice + swimming	Muscle-derived EVs(muscle-specific tracing)	Diverse functional miRNAs and proteins	Endothelial & Smoothmuscle cells	Inhibition of Endothelial Inflammation& adhesion molecules	Alleviated atherosclerosis;reduced plaque area; enhanced stability.	(75)

sEV, small extracellular vesicle; EVs, extracellular vesicles; SkM-EVs, skeletal muscle-derived EVs; miRNA, microRNA; HSP70, heat shock protein 70; MMP9, matrix metalloproteinase 9; HUVECs, human umbilical vein endothelial cells; ROS, reactive oxygen species; PGC-1α, peroxisome proliferator-activated receptor-gamma coactivator-1 alpha.

#### 4.1.1 Targeting apoptotic cascades

Exercise interventions systematically alter both the quantity and the molecular composition of circulating exosomes, making them potent anti-apoptotic agents. For instance, demonstrated a compelling cross-species effect: plasma exosomes isolated from both elite human rowers (following 1-year professional training) and mice subjected to a 4-week swimming regimen exhibited a significant upregulation of miR-342-5p (74). Upon internalization by cardiomyocytes, this exosomal miRNA directly targets and inhibits Caspase 9 and Jnk2 (96). By effectively blocking this mitochondria-dependent apoptotic pathway, exercise-derived EVs significantly reduced myocardial ischemic injury and preserved cardiac function (97).

Beyond miRNA-mediated epigenetic regulation, exercise-mobilized EVs also deliver protective protein cargos. Bei et al. (98) observed that a 3-week swimming protocol in mice not only increased the overall number of circulating EVs but also enriched them with Heat Shock Protein 70 (HSP70) and other vital proteins (98). When internalized by cardiomyocytes, these EVs triggered the activation of canonical pro-survival kinase cascades, including ERK1/2, p38 MAPK, and Akt signaling (99). This multidimensional signaling network significantly attenuated myocardial ischemia-reperfusion (I/R) injury, reduced infarct size, and fundamentally inhibited cardiomyocyte apoptosis (100).

#### 4.1.2 Reversing myocardial fibrosis

In the context of the aging and metabolically compromised heart, fibrosis dictates myocardial stiffness and diastolic dysfunction (101). Exercise-derived exosomes offer a targeted therapeutic strategy to reprogram ECM remodeling. Chaturvedi et al. (102) investigated this in a db/db diabetic mouse model paired with an 8-week swimming intervention (102). The researchers identified a specific cluster of miRNAs (miR-455, miR-29b, miR-323-5p, and miR-466) that were markedly upregulated in post-exercise serum and heart tissue exosomes (103). Functionally, these enriched miRNAs acted synergistically within cardiomyocytes to orchestrate the targeted inhibition of Matrix Metalloproteinase 9 (MMP9) expression (104). The suppression of MMP9 signaling directly attenuated diabetic myocardial fibrosis and facilitated beneficial ECM remodeling (105).

Collectively, these findings underscore that exercise orchestrates a systemic release of rejuvenating EVs. By delivering specific miRNAs (e.g., miR-342-5p, miR-29b) and stress-response proteins (e.g., HSP70) directly to cardiomyocytes, these vesicular carriers effectively dismantle apoptotic cascades and halt profibrotic signaling, offering molecular resilience to the aging heart (106).

### 4.2 Restoration of cardiac microcirculation: endothelial rejuvenation

The structural and functional integrity of the coronary microcirculation inevitably deteriorates with advancing age (107). This vascular aging is characterized by endothelial senescence, capillary rarefaction, and increased susceptibility to atherosclerosis, which collectively compromise oxygen and nutrient delivery to the myocardium (108). Exercise counteracts these age-related microvascular deficits by utilizing skeletal muscle-derived extracellular vesicles (SkM-EVs) as pivotal endocrine and paracrine vehicles to orchestrate endothelial rejuvenation (Table 2) (109).

#### 4.2.1 Stimulating angiogenesis and neutralizing oxidative stress

A primary mechanism by which exercise revives the aging cardiac microvasculature is the stimulation of angiogenesis coupled with the mitigation of endothelial oxidative stress. Nie et al. (71) delineated this pathway by demonstrating that exosomes secreted from differentiated C2C12 myotubes are highly enriched with miR-130a (71). Upon internalization by human umbilical vein endothelial cells (HUVECs), this specific microRNA cargo finely tunes ROS-mediated NF-*κ*B signaling pathways. This targeted epigenetic modulation significantly augments endothelial cell proliferation, migration, and tube formation, thereby promoting angiogenesis.

Complementing this miRNA-driven mechanism, SkM-EVs also serve as vital carriers for therapeutic messenger RNAs. Utilizing a PGC-1*α* overexpression model in primary human myotubes to mimic the physiological adaptations of exercise, Kargl et al. (70) revealed that the secreted SkM-EVs are packed with a potent antioxidant mRNA payload, notably including SOD2, Nrf2, and GPx (70). The direct transfer of these transcripts into endothelial cells substantially bolstered intracellular antioxidant capacity, effectively neutralizing reactive oxygen species (ROS). Consequently, this exosome-mediated redox buffering not only promoted cellular angiogenesis but also fundamentally resisted oxidative stress-induced endothelial senescence, indirectly but crucially preserving coronary microcirculation (110).

#### 4.2.2 Attenuating endothelial inflammation and atherosclerosis

Beyond fostering capillary networks, exercise-mobilized SkM-EVs exert profound protective effects against macrovascular pathologies that plague the aging cardiovascular system. Wang et al. (75) provided compelling *in vivo* evidence using an ApoE-/- mouse model of atherosclerosis subjected to a swimming regimen (75). By employing a skeletal muscle-specific promoter labeling technique, the researchers traced muscle-derived exosomes to their target sites in endothelial and smooth muscle cells. The integration of these EVs delivered a diverse repertoire of functional miRNAs and proteins that synergistically suppressed endothelial inflammation and downregulated the expression of detrimental adhesion molecules. Phenotypically, this exercise-driven “muscle-vessel” crosstalk markedly alleviated the atherosclerotic burden, reduced total plaque area, and critically enhanced plaque stability.

In summary, skeletal muscle-derived EVs function as multifaceted regulators of vascular homeostasis. By delivering specific pro-angiogenic miRNAs (miR-130a), antioxidant mRNAs, and anti-inflammatory cargos, exercise-rejuvenated SkM-EVs systematically restore endothelial vitality and microcirculatory networks, providing a robust defense against cardiac aging.

## 5 Clinical translation: muscle-derived EVs as biomarkers and therapeutics

As the cardioprotective mechanisms of exercise-derived SkM-EVs become increasingly elucidated, translating these biological insights into clinical practice represents the next major frontier. Building upon the macroscopic networks delineated in our Stage 3 data synthesis, the therapeutic targeting of the “muscle-heart” crosstalk must move beyond single-target pharmacology. As extracted in Table 1, the Stage 3 matrix aggregated overlapping signaling hubs (e.g., PI3 K/Akt) and multi-organ synergistic effects. This systems-level evidence supports two distinct translational avenues: utilizing EV cargos as non-invasive biomarkers for precision exercise prescription, and engineering exogenous EVs as “exercise mimetics” for patients incapable of physical exertion (34, 85).

### 5.1 Liquid biopsy: SkM-EVs as prognosticators for exercise responsiveness

Older adults show marked heterogeneity in their cardiovascular and metabolic responses to exercise, highlighting the need for precise prognostic biomarkers. Circulating SkM-EV miRNAs have emerged as highly sensitive “liquid biopsies” reflecting systemic metabolic health and musculoskeletal crosstalk (33). Miyamoto et al. (111) utilized exosomal multi-omics to demonstrate that EV-mediated signals directly correlate with early cardiac sympathetic denervation in Parkinson's disease, highlighting EVs as a diagnostic barometer for disrupted organ crosstalk (111).

In the context of exercise interventions, recent human cohort studies have solidified the predictive value of EVs. O'Bryan et al. (64) identified specific exosomal miRNA modules (e.g., miR-362-3p, miR-503-5p) in older adults undergoing resistance training that accurately predict muscle hypertrophic gains and anti-inflammatory adaptations (64). Furthermore, leveraging high-resolution flow cytometry, Zhang et al. (23) recently revealed that high-volume exercise robustly upregulates specific EV subpopulations negatively associated with insulin resistance (HOMA-IR) in older adults (88). These key findings are supported by a broader body of literature consistently demonstrating that exercise dynamically modulates circulating MyomiRs (miR-1, miR-133a, miR-203a-3p) to reflect systemic adaptations against metabolic dysfunction, atrophy, and aging (51, 60, 101).

### 5.2 “exercise mimetics”: nanotherapeutics for the frail heart

**Figure 4:**
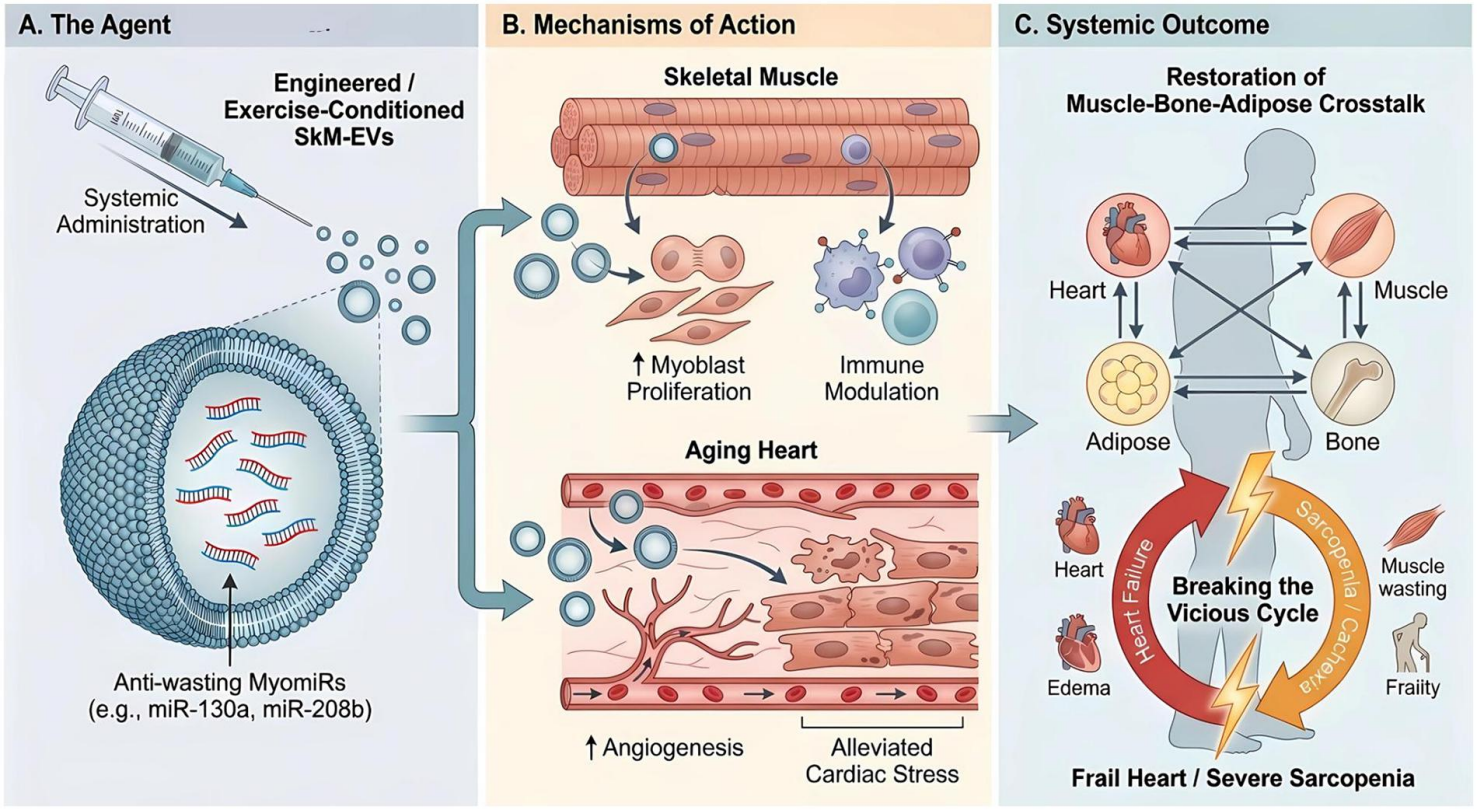
Conceptual framework of SkM-EVs as “exercise mimetics” for the frail cardiovascular aging population. **(A)** The Agent: For frail patients physically incapable of exercising, engineered or exercise-conditioned skeletal muscle-derived EVs (SkM-EVs) are utilized as cell-free therapeutics. These vesicles are loaded with anti-wasting MyomiRs (e.g., miR-130a, miR-208b) and delivered via systemic administration. **(B)** Mechanisms of Action: At the cellular level, the targeted delivery of these EVs exerts dual protective effects. In skeletal muscle, they promote myoblast proliferation and modulate the local immune microenvironment; simultaneously, in the aging heart, they induce angiogenesis and alleviate cardiac stress. **(C)** Systemic Outcome: Ultimately, this nanotherapeutic strategy acts as an artificial “exercise mimetic.” It restores the disrupted muscle-bone-adipose crosstalk and effectively breaks the vicious cycle between progressive heart failure and severe sarcopenia, achieving systemic rejuvenation without requiring actual physical exertion. (Created with BioRender.com).

In cardiovascular aging, a fundamental clinical paradox exists: the frail patients who most need exercise are often physically incapable of it (49). Consequently, utilizing SkM-EVs as cell-free “exercise mimetics” represents a paradigm-shifting therapeutic strategy (87). As systematically conceptualized in Figure 4, this nanotherapeutic approach encompasses a precise trajectory from systemic administration to multi-organ rejuvenation (36).

Following the systemic delivery of these agents (Figure 4A), the downstream outcomes reflect the macroscopic synergistic networks extracted in Stage 3. Specifically, this EV-mediated “polypharmacy” effect operates on multiple integrated fronts, which are systematically mapped out in Figure 4's translational framework (from the agent to systemic outcomes):

(1) The Therapeutic Agent and Local Muscle Rescue (Corresponding to Figure 4A,B): Rescuing skeletal muscle mass is a prerequisite for alleviating cardiac stress (112). Systemic administration of specialized EVs directly promotes myoblast proliferation and modulates local immune responses (62, 63).
(2) Multi-Organ Crosstalk Restoration (Corresponding to Figure 4C): By delivering EVs loaded with natural anti-wasting MyomiRs (e.g., miR-130a, miR-208b)—cargos systematically identified in our matrices—researchers can artificially restore the muscle-bone-adipose interactions lost in cachexia (61, 100).
(3) Targeted Cardiac Alleviation (The Final Systemic Outcome):Ultimately, for the aging heart, injecting these engineered or exercise-conditioned EVs simulates the systemic protective secretome of physical training, breaking the vicious cycle of frailty without requiring actual physical exertion (100).

### 5.3 Translational hurdles and rigorous methodological standardization

Moving forward, the field should overcome the limitations of standard ultracentrifugation by adopting rigorous density gradient methods and single-vesicle flow cytometry (54). Additionally, addressing the biodistribution challenges—whereby intravenously injected EVs are primarily sequestered by the liver and lungs before reaching the heart or muscle—requires the development of advanced cardiac-targeting peptides (89). Finally, establishing large-scale, multi-center human cohorts rather than relying solely on rodent models is imperative to validate the safety and pharmacokinetics of SkM-EV therapeutics in the aging population (23).

## 6 Translational potential and clinical challenges

### 6.1 Exosomes as liquid biopsies: guiding personalized “exercise prescriptions”

The paradigm of geriatric sports cardiology is undergoing a fundamental shift from generic physical activity recommendations to precision medicine (3). Central to this transition is the utilization of circulating SkM-EVs as highly sensitive, dynamic “liquid biopsies.” Because SkM-EVs traverse the systemic circulation to mediate the “muscle-heart” crosstalk, their molecular payload offers a real-time, non-invasive window into the biological age of the musculoskeletal system and its concurrent impact on the myocardium (24).

Based on the bimodal inter-organ communication network established in our review, SkM-EVs harbor a dual biomarker potential. In the pathological baseline of sarcopenia and physiological frailty, the systemic circulation is characterized by an overrepresentation of senescence-associated secretory phenotype (SASP)-related EVs (7). Specifically, the enrichment of myokines such as miR-34a—which actively drives cellular senescence—and the release of mitochondrial-derived vesicles (MDVs) containing damage-associated molecular patterns (DAMPs) serve as robust early warning signals for impending myocardial dysfunction and inflammaging (13). Conversely, mechanical loading through structured exercise fundamentally remodels this vesicular cargo into a cardioprotective profile (16). As evidenced by multiple *in vivo* models (Table 2), effective exercise interventions trigger the release of “exerkines,” characterized by the dramatic upregulation of cardioprotective miRNAs (e.g., anti-apoptotic miR-342-5p, anti-fibrotic miR-455/miR-29b clusters) and the direct transfer of antioxidant mRNAs (SOD2, Nrf2) and chaperone proteins (HSP70) (70, 71). Consequently, calculating the stoichiometric ratio between senescence-associated EV-miRNAs (e.g., miR-34a) and exercise-rejuvenated EV-miRNAs (e.g., miR-342-5p) could provide clinicians with a quantifiable “molecular index” of sarcopenic cardiomyopathy risk (66).

More importantly, this liquid biopsy approach addresses a critical hurdle in geriatric rehabilitation: determining the optimal “dose” of physical exertion. For older adults with preexisting cardiovascular vulnerabilities or severe muscle wasting, excessive mechanical stress may paradoxically exacerbate tissue damage, prompting the release of pro-inflammatory SkM-EVs rather than regenerative ones (42). By continuously monitoring the dynamic shifts in EV payloads, clinicians can titrate personalized “exercise prescriptions” (adjusting the modality, intensity, and duration, such as balancing aerobic endurance with resistance training) (41). For instance, reaching a systemic threshold where endothelial-rejuvenating cargos like miR-130a peak—without triggering pathological mechanotransduction signals—would signify the optimal therapeutic window. Ultimately, integrating SkM-EV miRNA profiling into routine clinical assessments will bridge the gap between empirical physiotherapy and precision molecular medicine, ensuring that exercise serves as a precisely targeted physiological intervention for the aging heart (43).

### 6.2 Pharmacological interventions: developing SkM-EV-based “exercise mimetics”

While the pleiotropic benefits of mechanical loading are undeniable, a significant proportion of the geriatric population—particularly those with advanced sarcopenia, severe osteoarthritis, or decompensated heart failure—exhibits absolute or relative contraindications to rigorous exercise (2). This clinical reality necessitates the development of pharmacological “exercise mimetics.” (1) Skeletal muscle-derived extracellular vesicles (SkM-EVs), owing to their low immunogenicity, innate ability to cross biological barriers, and exceptional cargo stability, emerge as ideal candidates for next-generation cell-free therapeutics (90). Proof-of-concept studies extracted in our review (Table 2), such as the PGC-1*α* overexpression model by Kargl et al., demonstrate that mimicking exercise signaling *in vitro* can generate SkM-EVs robustly enriched with antioxidant mRNAs (e.g., SOD2, Nrf2) (70). By leveraging cutting-edge nanotechnology, researchers can systematically harvest these natural exercise-conditioned EVs, or engineer naive exosomes to carry defined cardioprotective payloads identified in this review (such as anti-apoptotic miR-342-5p or pro-angiogenic miR-130a) (52). Theoretically, this approach holds the potential to deliver a highly targeted pharmacological exercise mimetic directly to the ischemic or aging myocardium (113).

Parallel to delivering protective payloads, therapeutic strategies should concurrently neutralize the pathological inter-organ crosstalk emanating from the senescent musculoskeletal system (27). As highlighted in our pathological baseline synthesis, aged skeletal muscle acts as a systemic endocrine disruptor, continuously shedding EVs laden with pro-senescence factors (e.g., miR-34a, miR-125a-5p) and inflammatory myokines (14, 28). Consequently, targeted pharmacological interventions aiming to disrupt this toxic communication network hold profound clinical promise. For instance, the systemic administration of specific AntagomiRs designed to silence circulating senescent myomiRs could prevent age-related myocardial fibrosis (4). Furthermore, utilizing targeted senolytic therapies to selectively clear senescent satellite cells and atrophic myofibers could effectively “cap” the source of SASP-related EVs, thereby uncoupling the degenerating muscle from the vulnerable heart.

### 6.3 Methodological hurdles and future clinical directions

Despite the compelling mechanistic evidence supporting the skeletal muscle-heart axis, several formidable methodological hurdles should be surmounted to facilitate true clinical translation.

The first major challenge lies in the isolation, standardization, and tissue-specific tracing of circulating EVs. The systemic EV pool is highly heterogeneous, comprising vesicles originating from the liver, endothelium, adipose tissue, and hematopoietic cells (10). While conventional isolation techniques (e.g., ultracentrifugation or size-exclusion chromatography) yield high EV quantities, they cannot distinguish muscle-derived vesicles from others (11). To definitively map the “muscle-to-heart” trajectory, future basic research should pivot toward advanced genetic lineage tracing. For instance, the methodology employed by Wang et al. (Table 2), utilizing skeletal muscle-specific promoter labeling in transgenic murine models, represents the gold standard for tracking endogenous EV biodistribution (46). Translating this precision into human studies requires the identification of highly specific, universally accepted SkM-EV surface markers (beyond classical CD81/CD9/CD63) to accurately capture the muscle-derived fraction from human plasma (47).

The second obstacle pertains to the pharmacokinetics and targeted delivery of exogenous EV therapeutics. As briefly noted, systemically administered “exercise-mimetic” EVs are susceptible to rapid clearance by the mononuclear phagocyte system, predominantly accumulating in the liver, lungs, and spleen (72). Achieving a therapeutically viable concentration in the aging myocardium requires bypassing this physiological “sink.” Future bioengineering endeavors should explore the functionalization of EV membranes with cardiac-homing peptides (e.g., ischemic myocardium-targeted peptides) or the integration of EVs with biocompatible hydrogels for localized epicardial delivery, thereby maximizing myocardial retention and minimizing systemic off-target effects (53).

Finally, a critical translational gap exists between current pre-clinical models and the human geriatric reality. A profound observation from our data synthesis is that the majority of mechanistic insights are derived from artificial murine paradigms (e.g., forced swimming, lifelong treadmill running) or *in vitro* myotube cultures (e.g., C2C12 cells). These models do not perfectly recapitulate the complex, multi-morbidity landscape of frail human aging. Furthermore, available human data predominantly focus on acute exercise bouts rather than chronic adaptations (21). Therefore, future clinical directions should prioritize large-scale, multi-center, longitudinal human cohorts. Integrating multi-omics (transcriptomics and proteomics) to profile plasma SkM-EVs in older adults undergoing standardized aerobic and resistance training will be imperative to validate the safety, efficacy, and prognostic value of exercise-conditioned EVs (48, 86).

## 7 Conclusion and future perspectives

Cardiac aging is no longer seen as an isolated myocardial event; it is a systemic consequence of inter-organ decline. As synthesized in this review, the “muscle-heart” crosstalk mediated by SkM-EVs serves as a critical bimodal axis in cardiovascular health. During the progression of sarcopenia and physiological aging, the skeletal muscle acts as a systemic endocrine disruptor. It continuously sheds SASP-associated SkM-EVs, heavily laden with pathological myomiRs and mitochondrial DAMPs, which actively propagate inflammaging, endothelial senescence, and myocardial fibrosis.

Conversely, rigorous mechanical loading potently resets this system. Exercise rejuvenates this disrupted communication network by orchestrating the release of “exerkines”—specifically, SkM-EVs enriched with a pleiotropic network of cardioprotective transcripts. These molecular messengers directly neutralize cardiac oxidative stress, inhibit apoptosis, and promote microvascular angiogenesis. Therefore, physical training represents a highly efficacious non-pharmacological strategy to restore musculoskeletal-cardiovascular homeostasis.

Looking toward the future, deciphering the exosomal language between the muscle and the heart opens unprecedented avenues for geriatric cardiology. The immediate clinical horizon involves leveraging circulating SkM-EV profiles as dynamic “liquid biopsies” to gauge systemic biological age, thereby enabling the titration of precision exercise prescriptions for the elderly. Concurrently, the ultimate translational frontier lies in bioengineering. By conquering the existing hurdles of organ-specific biodistribution and EV standardization, the development of targeted “exercise mimetics”—next-generation nanotherapeutics delivering exercise-conditioned EV payloads directly to the failing heart—will become a clinical reality. Ultimately, shifting our therapeutic focus from isolated organs to dynamic inter-organ networks will empower us not merely to treat age-related cardiovascular diseases, but to proactively compress morbidity and significantly extend the human healthspan.
